# Strategies for genotype imputation in composite beef cattle

**DOI:** 10.1186/s12863-015-0251-7

**Published:** 2015-08-07

**Authors:** Tatiane C. S. Chud, Ricardo V. Ventura, Flavio S. Schenkel, Roberto Carvalheiro, Marcos E. Buzanskas, Jaqueline O. Rosa, Maurício de Alvarenga Mudadu, Marcos Vinicius G. B. da Silva, Fabiana B. Mokry, Cintia R. Marcondes, Luciana C. A. Regitano, Danísio P. Munari

**Affiliations:** Departamento de Ciências Exatas, UNESP - Univ Estadual Paulista “Júlio de Mesquita Filho”, Jaboticabal, SP Brazil; Departamento de Zootecnia, UNESP - Univ Estadual Paulista “Júlio de Mesquita Filho”, Jaboticabal, SP Brazil; Beef Improvement Opportunities, Guelph, ON Canada; University of Guelph, Guelph, ON Canada; Embrapa Southeast Livestock - Brazilian Corporation of Agricultural Research, São Carlos, SP Brazil; Embrapa Dairy Cattle - Brazilian Corporation of Agricultural Research, Juiz de Fora, MG Brazil; Department of Genetics and Evolution, Federal University of São Carlos, São Carlos, SP Brazil

**Keywords:** Canchim breed, Crossbred cattle, Genomic data, Low-density panel, Single nucleotide polymorphism

## Abstract

**Background:**

Genotype imputation has been used to increase genomic information, allow more animals in genome-wide analyses, and reduce genotyping costs. In Brazilian beef cattle production, many animals are resulting from crossbreeding and such an event may alter linkage disequilibrium patterns. Thus, the challenge is to obtain accurately imputed genotypes in crossbred animals. The objective of this study was to evaluate the best fitting and most accurate imputation strategy on the MA genetic group (the progeny of a Charolais sire mated with crossbred Canchim X Zebu cows) and Canchim cattle. The data set contained 400 animals (born between 1999 and 2005) genotyped with the Illumina BovineHD panel. Imputation accuracy of genotypes from the Illumina-Bovine3K (3K), Illumina-BovineLD (6K), GeneSeek-Genomic-Profiler (GGP) BeefLD (GGP9K), GGP-IndicusLD (GGP20Ki), Illumina-BovineSNP50 (50K), GGP-IndicusHD (GGP75Ki), and GGP-BeefHD (GGP80K) to Illumina-BovineHD (HD) SNP panels were investigated. Seven scenarios for reference and target populations were tested; the animals were grouped according with birth year (S1), genetic groups (S2 and S3), genetic groups and birth year (S4 and S5), gender (S6), and gender and birth year (S7). Analyses were performed using FImpute and BEAGLE software and computation run-time was recorded. Genotype imputation accuracy was measured by concordance rate (CR) and allelic R square (R^2^).

**Results:**

The highest imputation accuracy scenario consisted of a reference population with males and females and a target population with young females. Among the SNP panels in the tested scenarios, from the 50K, GGP75Ki and GGP80K were the most adequate to impute to HD in Canchim cattle. FImpute reduced computation run-time to impute genotypes from 20 to 100 times when compared to BEAGLE.

**Conclusion:**

The genotyping panels possessing at least 50 thousands markers are suitable for genotype imputation to HD with acceptable accuracy. The FImpute algorithm demonstrated a higher efficiency of imputed markers, especially in lower density panels. These considerations may assist to increase genotypic information, reduce genotyping costs, and aid in genomic selection evaluations in crossbred animals.

**Electronic supplementary material:**

The online version of this article (doi:10.1186/s12863-015-0251-7) contains supplementary material, which is available to authorized users.

## Background

The recent implementation of genomic selection in cattle breeding programs has allowed the rate of genetic progress to increase, especially in the dairy industry [1]. Selection based on genetic markers requires a large number of genotyped individuals and thousands of single nucleotide polymorphisms (SNP) scattered throughout the genome [2]. The improvement in accuracy of genomic selection in beef cattle, which often includes data from different breeds and crossbred animals, depends on conservation of linkage disequilibrium, consistency of the linkage phase between QTL (quantitative trait loci) and genetic markers across breeds, and similarity of QTL effects between breeds [3, 4].

In some dairy breeds, animals have been genotyped with 50,000 SNPs (50K). However, the 50K panel generally does not increase genomic selection accuracy in combined data from different breeds [1, 5]. According to de Roos et al. [6], more than 300,000 informative SNPs are required to detect conserved linkage disequilibrium and allow multi-breed genomic selection. High-density panels have higher coverage of SNPs in smaller genomic distances, greater linkage disequilibrium and conserved linkage disequilibrium across breeds, and are better for genomic selection and genome-wide association studies in beef cattle and crossbred animals [3, 7]. However, genotyping with high-density panels remains costly and can limit the number of animals used in genomic studies. An alternative that reduces these costs is genotype imputation [8, 9].

Genotype imputation is a method that allows for inferring the missing marker genotypes from individuals genotyped with low and medium density (LD) panels by using information from a reference population genotyped with high-density panels [10, 11]. This makes it possible to increase the genomic information and predict missing genotypes [7, 12], reduce genotyping costs and intensify genomic selection [13, 14], and combine data from different breeds [11, 15].

Imputation methods may be based on family information (using pedigree), thus using Mendelian segregation rules and linkage to predict genotypes, and/or on population-based information; wherein genotypes are predicted by means of linkage disequilibrium observed between markers in the reference population [11]. Imputation accuracy is influenced by several factors such as population structure, reference population size, the number of SNPs in the LD panel, marker frequency, relatedness between the reference and the target populations, and the imputation tools [10, 16, 17].

In Brazil, crossbreeding schemes have been used to develop composite breeds such as the Canchim, originating from alternate crosses between Charolais (*Bos taurus taurus*) and Zebu breeds (*Bos taurus indicus*) [18]. Generally, the final genetic composition of Canchim animals is 62.5 % Charolais and 37.5 % Zebu; however, different proportions of Charolais/Zebu genes may be present in Canchim animals due to the various mating schemes which have been used to expand the genetic base for this breed [19]. One such scheme produces the “MA” genetic group, which is the progeny of a Charolais sire mated with crossbred Canchim X Zebu cows. The expected proportion of genes for MA is approximately 65.6 % Charolais and 34.4 % Zebu.

In Brazilian beef cattle production, many animals result from crossbreeding between or within *Bos taurus taurus* and *Bos taurus indicus*. Therefore, genotype imputation in crossbred animals remains a challenging task; leading to the development of methodologies and imputation strategies that can maximize accuracy in the population of interest. The objective of this study was to evaluate the best fitting and most accurate imputation strategy for the MA genetic group and Canchim cattle.

## Methods

### Ethics statement

This study had the approval of the Embrapa Southeast Livestock Ethical Committee of Animal Use (CEUA- CPPSE), under protocol number 02/2009.

### Data set and genotype

The genomic database used in this study was provided by the Brazilian Corporation of Agricultural Research (Embrapa), located in São Carlos, SP, Brazil.

Four hundred animals, born between 1999 and 2005, were genotyped with the BovineHD BeadChip (Illumina, Inc., San Diego, CA) panel, consisting of 786,799 SNPs distributed throughout the genome. There were 205 females and 195 males in the data set. Approximately half of the animals (194) were from Embrapa, originating from 17 different bulls; 186 were Canchim and 8 were MA. The remaining animals were from farms located in São Paulo (38 Canchim and 9 MA) and Goiás (60 Canchim and 97 MA animals) states, and 1 Canchim bull and 1 Charolais bull that were parents of 7 and 14 genotyped individuals, respectively.

The pedigree relationship matrix of these animals consisted of 4,095 animals and the average inbreeding was equal to 0.02, calculated by the CFC program [20]. The Canchim animals were progeny from 40 Canchim bulls and presented an average relatedness of 0.005, while the MA animals were progeny from 10 Charolais bulls, and presented an average relatedness of 0.018.

The average linkage disequilibrium between adjacent markers in the original HD panel (Additional file 1) was calculated using the SNPPLD software [21], with r^2^ as the linkage disequilibrium measure [22].

### Data quality control

Only the autosomal chromosomes and SNPs with known positions in the UMD_3.1 bovine assembly map [23] were considered. Genotype quality control (QC) excluded SNPs with a call rate lower than 0.90, SNPs with deviations from the Hardy-Weinberg equilibrium (p < 10^−6^) as calculated by means of the Fisher’s Exact Test, SNPs with proportion of expected heterozygous higher than 0.85 [24], and SNPs with minor allele frequency (MAF) lower than 0.0025. For the QC of the samples, animals with a call rate lower than 0.90 were excluded from analysis. The final file contained 396 animals and 616,565 SNPs.

### Low and medium density SNP panels

The low and medium density panels were created by masking SNPs originally present in the Illumina® BovineHD SNP panel by selecting the markers in common with the Illumina® Bovine3K (3K), Illumina® BovineLD (6K), GeneSeek® Genomic Profiler (GGP) Beef LD (GGP9K), GGP Indicus LD (GGP20Ki), Illumina® BovineSNP50 version 2 (50K), GGP Indicus HD (GGP75Ki), and GGP Beef HD (GGP80K) (Table 1). The number of SNPs that remained after QC for the GGP75Ki (for indicine breeds) and GGP80K (for taurine breeds) may reflect the genetic composition of Canchim, because as previously mentioned, the contribution of the Charolais breed (taurine) is higher than the Zebu.

**Table 1:** Number of SNPs in common between LD^a^ panel and the HD^b^ panel

LD Panel	Label	SNPs in original LD panel	SNPs in common after QC^c^
Illumina® Bovine 3K	3K	2,900	2,341
Illumina® Bovine LD	6K	6,909	6,280
GGP Beef LD	GGP9K	8,762	7,548
GGP Indicus LD	GGP20Ki	19,721	14,305
Illumina® BovineSNP50	50K	54,609	38,802
GGP Indicus HD	GGP75Ki	74,085	50,038
GGP Beef HD	GGP80K	76,992	67,143

^a^LD: low-density, ^b^HD: high-density panel, ^c^QC: quality Control

### Genotype imputation

According to the possible situations, seven scenarios for reference and target populations were tested in order to identify the scenario that fit our data set and, as an extension, for composite beef cattle breeds (Table 2). Briefly, animals were grouped in scenarios considering birth year (S1), genetic groups (S2 and S3), genetic groups and birth year (S4 and S5), gender (S6), and gender and birth year (S7).

**Table 2:** Description of imputation scenarios and number of animals in reference^a^ and target^b^ population

Scenarios	Description	Number of animals			
		Charolais	Canchim	MA	Total
S1	Animals born prior to 2005^a^	1	184	68	253
	Animals born in 2005^b^	0	99	44	143
S2	All Canchim animals^a^	0	283	0	283
	All MA animals^b^	0	0	112	112
S3	All MA animals^a^	0	0	112	112
	All Canchim animals^b^	0	283	0	283
S4	All Canchim + MA animals born prior to 2005^a^	0	283	68	351
	MA animals were born in 2005^b^	0	0	44	44
S5	All MA + Canchim animals born prior to 2005^a^	0	184	112	296
	Canchim animals were born in 2005^b^	0	99	0	99
S6	All males^a^	1	128	63	192
	All females^b^	0	155	49	204
S7	All Males + Females born prior to 2005^a^	1	228	86	315
	Females born in 2005^b^	0	55	26	81

^a^Reference population; ^b^Target Population

The population genotype imputation was implemented using the FImpute v2.2 [25] and BEAGLE v3.3.2 software [26]. We used population-based imputation for both programs. The imputation accuracy was calculated by means of two criteria:

i. Concordance rate (CR) - The imputed markers were compared with the actual markers present in the original HD panel, and thus the proportion of genotypes that were imputed correctly or erroneously was calculated. The concordance rate represents the proportion of correctly imputed genotypes.

ii. Allelic r-squared correlation (allelic R^2^) - The allelic R^2^ is determined by the square of the correlation between the allele dosage of the most likely imputed genotype and the allele dosage of the true genotype [26].

The effect of genetic relatedness between the validation and reference animals (Table 3) on imputation accuracy was assessed by regressing the concordance rate on the maximum genomic relationship between each animal in the validation set and all the animals in the reference set [27]. The average genomic relationship (G) was calculated according to VanRaden [28]:

**Table 3:** Genomic relationship statistics between reference population and target population

Scenarios^a^	Genomic Relationship		
	Minimum	Mean	Maximum
S1	0.023	0.198	0.390
S2	0.010	0.050	0.220
S3	0.003	0.040	0.225
S4	0.028	0.193	0.330
S5	0.050	0.198	0.390
S6	0.090	0.210	0.409
S7	0.108	0.228	0.390

^a^As described in the section “Genotype imputation” of “Methods”

1 $$ G=\frac{MM\mathit{\hbox{'}}}{{\displaystyle \sum 2{p}_i\left(1-{p}_i\right)}} $$

in which *M* is the incidence matrix of markers whose elements in the *i*^*th*^ column are 0-2*p*_*i*_, 1-2*p*_*i*,_ and 2-2*p*_*i*_ for genotypes AA, AB and BB, respectively; *M’* is the transpose of the incidence matrix; and *p*_*i*_ is the frequency of allele B in the *i*^*th*^ marker.

## Results and discussion

### Imputation accuracy

**Fig. 1:**
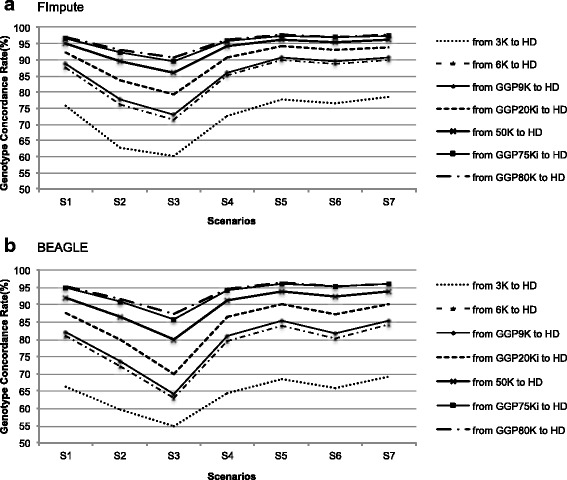
Genotype concordance rate using FImpute (**a**) and BEAGLE (**b**) software for all scenarios tested. S1: animals born prior to 2005 in reference population and in target population animals born in 2005; S2: Canchim animals in reference population and MA animals in target population; S3: MA animals in reference population and Canchim animals in target population; S4: all Canchim + MA animals born prior to 2005 in reference population and MA animals were born in 2005 in target population; S5: All MA + Canchim animals born prior to 2005 in reference population and Canchim animals were born in 2005 in target population; S6: all males in reference population and all females in target population; S7: All Males + Females born prior to 2005 in reference population and Females born in 2005 in target population

When the Flmpute software was used, the overall average imputation accuracy from LD to HD by concordance rate ranged from 60 to 98 %; and by the allelic R^2^ measure ranged from 0.33 to 0.96 (Table 4; Fig. 1). Using the BEAGLE software, the overall average imputation accuracy ranged from 55 to 96 % by CR and from 0.25 to 0.94 by the allelic R^2^ (Table 4). We found that when the CR is high, the allelic R^2^ value approaches this rate. The allelic R^2^ value is smaller than the CR because this method has no relationship to MAF [7, 29, 30].

**Table 4:** Imputation accuracy from low-density panel to high-density panel using FImpute and BEAGLE software

Scenarios^a^	LD panel	FImpute		BEAGLE	
		CR%^b^	R^2c^	CR%^b^	R^2c^
S1	3K	75.70	0.59	66.27	0.44
	6K	87.72	0.79	80.79	0.68
	GGP9K	88.64	0.81	82.19	0.70
	GGP20Ki	92.43	0.87	87.50	0.71
	50K	95.20	0.92	92.14	0.87
	GGP75Ki	96.68	0.94	95.03	0.92
	GGP80K	96.96	0.95	95.26	0.92
S2	3K	62.86	0.37	59.73	0.33
	6K	76.17	0.58	72.23	0.58
	GGP9K	77.54	0.61	73.78	0.55
	GGP20Ki	83.61	0.71	79.75	0.65
	50K	89.55	0.82	86.66	0.77
	GGP75Ki	92.48	0.87	90.85	0.84
	GGP80K	93.24	0.88	91.51	0.85
S3	3K	60.21	0.33	54.83	0.25
	6K	71.46	0.51	63.00	0.38
	GGP9K	72.93	0.54	64.15	0.40
	GGP20Ki	79.19	0.65	69.91	0.49
	50K	85.92	0.76	79.95	0.66
	GGP75Ki	89.54	0.82	85.79	0.76
	GGP80K	90.60	0.84	87.35	0.79
S4	3K	72.75	0.53	64.55	0.40
	6K	85.17	0.74	79.32	0.65
	GGP9K	86.12	0.76	80.85	0.67
	GGP20Ki	90.60	0.84	86.55	0.77
	50K	94.12	0.90	91.24	0.85
	GGP75Ki	95.94	0.93	94.36	0.90
	GGP80K	96.28	0.93	94.53	0.91
S5	3K	77.74	0.62	68.57	0.47
	6K	89.84	0.83	83.86	0.73
	GGP9K	90.67	0.84	85.23	0.75
	GGP20Ki	94.15	0.94	90.23	0.84
	50K	96.36	0.90	93.90	0.90
	GGP75Ki	97.55	0.96	96.10	0.94
	GGP80K	97.74	0.96	96.30	0.94
S6	3K	76.52	0.60	65.80	0.43
	6K	88.71	0.81	80.35	0.67
	GGP9K	89.56	0.82	81.71	0.70
	GGP20Ki	93.13	0.88	87.33	0.80
	50K	95.60	0.93	92.23	0.87
	GGP75Ki	96.98	0.95	95.25	0.92
	GGP80K	97.19	0.95	95.40	0.92
S7	3K	78.69	0.64	69.06	0.48
	6K	89.98	0.83	84.16	0.73
	GGP9K	90.76	0.85	85.42	0.76
	GGP20Ki	94.06	0.90	90.20	0.84
	50K	96.27	0.94	93.82	0.90
	GGP75Ki	97.47	0.96	96.04	0.93
	GGP80K	97.66	0.96	96.20	0.94

^a^As described in the section “Genotype imputation” of “Methods,^b^CR = Concordance Rate, ^c^R^2^: Allelic R square

Ventura et al. [13], who imputed genotypes from 6K to 50K in Canadian crossbreed beef cattle, found overall average concordance rates ranging from 54 to 97 % (using FImpute) and from 54 to 96 % (using BEAGLE). Piccoli et al. [31] found results similar to ours when studying Brazilian Braford and Hereford beef cattle and imputing from various low-density panels to HD. Carvalheiro et al. [9], working with Nelore animals, found concordance rates of 97 and 99 % when using the GGP20Ki and GGP75Ki for genotype imputation to the HD panel. The imputation accuracy is lower in beef cattle populations than in dairy cattle populations due limited number of animals in the genotyped reference population, the larger number of effective ancestors, and the lower relatedness between reference and target populations [3].

The average gain in the CR from the GGP75Ki and GGP80K SNP panels to the HD panel, when compared to the 3K to HD, was 24 % for FImpute and 29 % for BEAGLE. The rate of correctly imputed genotypes increased as the number of SNP markers present in each of the LD panels increased (Fig. 1). The prediction of haplotypes and the linkage disequilibrium between markers are affected when the genotyping panel is composed by few SNPs; thus, the density of the tested LD panel is an important factor affecting imputation accuracy [10, 32]. Studies have shown that the accuracy of the predicted genomic value decreases with increasing imputation error rates [33, 34]. Furthermore imputation errors can lead to bias in predicting breeding values [35, 36].

The most suitable LD panels were the 50K, GGP80K, and GGP75Ki, because they had the highest imputation accuracy for genotype imputation in Canchim cattle. Although the GGP75Ki and GGP80K panels have been developed for *Bos taurus indicus* and *Bos taurus taurus*, respectively*,* and they have different markers, no differences in the average imputation accuracy were observed. As the Canchim is a composite breed, we can suggest that this result reflects the genetic background from both Taurine and Indicine breeds.

**Fig. 2:**
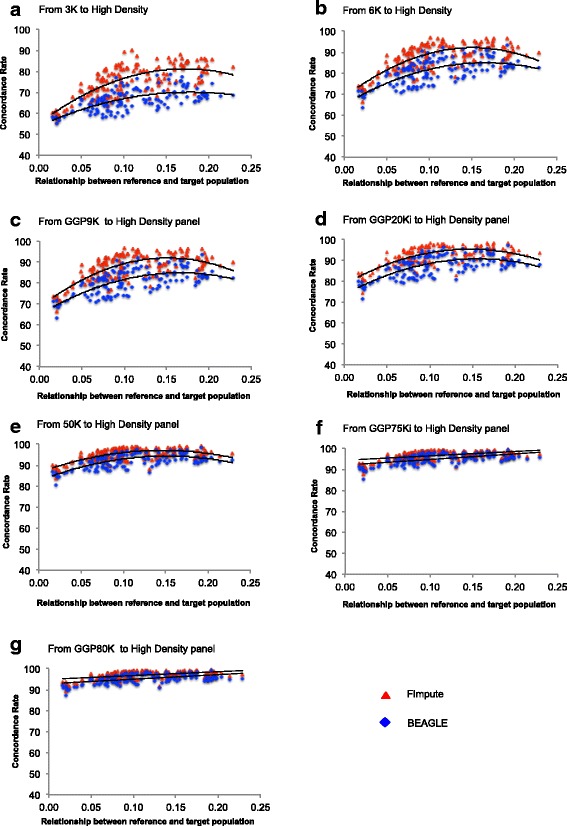
Average relationship between reference and target population. Figure 2 shows average relationship between reference and target population considering scenario S1 (animals grouped considering birth year) for genotype imputation from panels 3K (**a**), 6K (**b**), GGP9K (**c**), GGP20Ki (**d**), 50K (**e**), GGP 75Ki (**f**), and GGP80K (**g**) to High Density (HD) panel. Regression equation was significant (p < 0.01) for all panels

Target population individuals presenting a higher average relatedness to the reference population had higher concordance rates. We observed a curvilinear increase (p < 0.01) in the concordance rate when the relatedness between reference and target individuals increased (Fig. 2). The imputation accuracy from lower-density panels (3K, 6K, GGP9K, GGP20Ki) to HD was affected by the low relatedness between reference individuals and target individuals; while the imputation accuracy from higher-density panels (50K, GGP75Ki, GGP80K) to HD had better results. Zhang and Druet [37] and Carvalheiro et al. [9] obtained results similar to ours and found better imputation accuracy from lower-density panels to HD in related individuals.

**Fig. 3:**
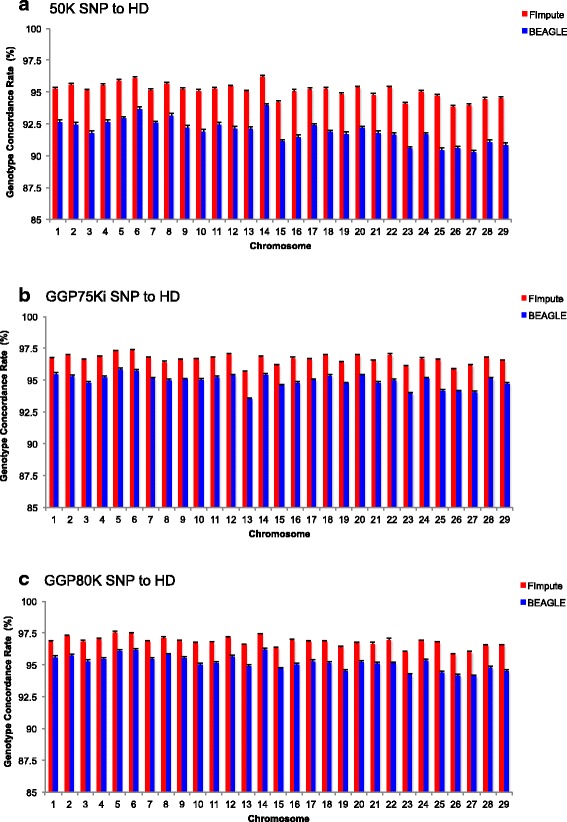
Genotype concordance rate by chromosome using FImpute and BEAGLE software. Considering individuals grouped by birth year (S1) from 50K SNP to HD (**a**), GGP 75Ki SNP to HD (**b**), and GGP80K SNP to (**c**)

Because there is a different coverage of SNPs per chromosome, the panels showed different imputation errors per chromosome (Fig. 3). Chromosome 27 showed the lowest imputation accuracy from 50K to GGP80K panels to HD, while in the GGP75Ki panel, chromosome 13 had the lowest imputation accuracy. In the 50K and GGP80K panels, chromosome 13 had a greater number of SNPs (1330 SNPs and 2202 SNPs, respectively) compared to the GGP75Ki panel (1273 SNPs), providing further information for haplotype inferences. In addition, chromosome 13 showed lower average linkage disequilibrium between adjacent markers for the HD panel (S1 Table). Imputation differences may occur due to the difficulties in correctly imputing the initial and end regions of chromosomes [31], consequently shorter chromosomes presented less accurately imputed alleles. Pausch et al. [17], imputing from 50K to 777K SNP panel in Fleckvieh cattle, found higher and lower accuracies on BTA1 and on BTA25, respectively. Moreover, low imputation accuracies on chromosomes can be due to mapping errors [38].

### Scenarios of reference and target populations for genotype imputation

In practical terms and for future applications, scenario S1 (Table 2) was suggested as the most appropriate for genomic studies in Canchim cattle, because the reference population includes older animals, while younger animals are included in the target population. The concordance rate from 50K, GGP75Ki and GGP80K to HD (Table 4) was acceptable for scenario S1 (overall average 95.2 %). Thus, young Canchim candidates could be genotyped with low-density panels (50K, GGP75Ki and, GGP80K), thereby reducing costs and enabling the breeding programs to include genotype data for genomic selection.

The imputation accuracy when using Canchim animals as the reference population and MA animals as the target population (S2) was better than the opposite situation (S3). Using a larger number of individuals in the reference population could aid in estimating more reliable haplotypes [10, 13, 34] and therefore present better imputation accuracy. Another important issue was that scenarios S2 and S3 had the lowest average relatedness (0.05 and 0.04 respectively) between reference and target populations (Table 3). Thus, in order to increase imputation accuracy, it is important to maintain the relatedness between reference and target populations [3].

Scenario S5 presented the highest CR. The possible reasons for this result were that S5 presents a balanced number of Canchim and MA animals in the reference population, a high number of animals in the reference population, both males and females were included in the reference and target population, the reference population considered varied ages, and the mean genomic relatedness was the third highest. Despite of a very similar construction, the S4 imputation accuracy was lower than scenario S5. The main difference between both scenarios was the mean genomic relatedness between reference and target population.

The gender division (scenarios S6 and S7) showed that the imputation of female genotypes could be carried out using only the males in a reference population. Scenarios S6 and S7 had the highest mean genomic relatedness between reference and target populations, which may have contributed to the imputation accuracy. Genotype imputation using females genotyped with low-density panels could be an appropriate strategy for large-scale female selection [39].

### FImpute versus BEAGLE

**Fig. 4:**
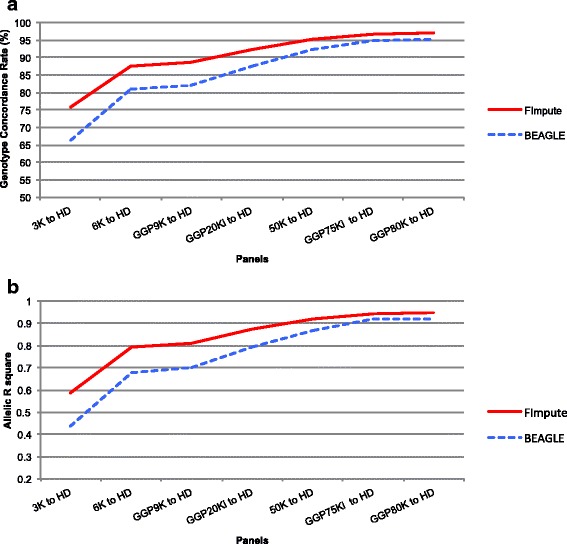
Concordance Rate (**a**) and Allelic R-square (**b**) using FImpute and BEAGLE software. Considering the scenario S1 (individuals grouped by birth year)

FImpute demonstrated better imputation performance, especially for low-density panels (3K, 6K, GGP9K, and GGP20Ki) (Fig. 4). Moreover, low gains in imputation accuracy for 50K, GGP75Ki and GGP80K were observed. The FImpute algorithm reduces imputation error because it uses overlapping windows to identify long identity-by-descent segments, which facilitates the identification of haplotypes in panels with few markers. The BEAGLE software was developed for human populations and requires more complex algorithms due to population structure, as well as greater computational demand for haplotype construction. In our study, FImpute software reduced run-time from 20 to 100 times when compared to BEAGLE software. The issue of computational demand is very important due to the increasing number of animals being genotyped. Ventura et al. [13] reported run-time reductions of 13 to 52 times for genotype imputation when FImpute was compared to BEAGLE. Although imputation has great advantages, large-scale computational resources are required and imputation accuracy must be evaluated.

## Conclusion

Low-density panels possessing at least 50 thousands markers (50K, GGP80K, and GGP75Ki) are suitable for genotype imputation to HD with acceptable accuracy. Canchim and MA animals from both sexes should be considered in the reference population. The scenario with the MA and Canchim animals born prior to 2005 in the reference population and the young Canchim animals in the target population was the best fitting to our data; however, it would be more practical to genotype young males and females with low-density panels (50K, GGP75Ki, and GGP80K) as the target population, and maintain older animals in the reference population (S1). The FImpute algorithm demonstrated higher efficiency of imputed markers (best accuracy and lowest run-time), especially in lower density panels (3K, 6K, GGP9K, and GGP20Ki). These considerations may assist in increasing genotypic information, decrease the run-time of analyses, reduce genotyping costs, and aid in genomic selection evaluations in Canchim cattle.

### Availability of supporting data

The genomic data used in this study is available upon request from Dr. Luciana Correia de Almeida Regitano (Embrapa Livestock Southeast - Rodovia Washington Luiz, km 234, São Carlos, São Paulo, 13560–970, Brazil, Tel: 55 16 3411–5600).

## Additional file

Additional file 1:
**Average linkage disequilibrium (r2) by chromosome between adjacent markers.**

## Abbreviations

3K: Illumina-Bovine3K SNP panel
6K: Illumina-BovineLD SNP panel
GGP: GeneSeek Genomic Profiler SNP panel
GGP9K: GeneSeek Genomic Profiler BeefLD SNP panel
GGP20Ki: GeneSeek Genomic Profiler IndicusLD SNP panel
50K: Illumina-BovineSNP50 SNP panel
GGP75Ki: GeneSeek Genomic Profiler IndicusHD SNP panel
GGP80K: GeneSeek Genomic Profiler BeefHD SNP panel
HD: Illumina-BovineHD SNP panel
S1: Scenario 1: animals were grouped considering birth year
S2: Scenario 2: animals were grouped considering genetic groups
S3: Scenario 3: animals were grouped considering genetic groups
S4: Scenario 4: animals were grouped considering genetic groups and birth year
S5: Scenario 5: animals were grouped considering genetic groups and birth year
S6: Scenario 6: animals were grouped considering gender (S6)
S7: Scenario 7: animals were grouped considering gender and birth year
CR: Concordance rate
R^2^: Allelic R square
SNP: Single nucleotide polymorphisms
QTL: Quantitative trait loci
LD: Low and medium density panels
MA: Progeny of a Charolais sire mated with crossbred Canchim X Zebu cows
r^2^: Linkage disequilibrium measure
QC: Quality control
MAF: Minor allele frequency
G: Average genomic relationship
