# Supplementary material for: Strategies for genotype imputation in composite beef cattle
Source: BMC Genet. 2015 Aug 7;16:99. doi: 10.1186/s12863-015-0251-7 (PMC4527250; doi:10.1186/s12863-015-0251-7)
Supplement: Additional file 1: — Average linkage disequilibrium (r2) by chromosome between adjacent markers. [file 12863_2015_251_MOESM1_ESM.docx]

**Additional File 1.** Average linkage disequilibrium (r^2^) by chromosome between adjacent markers.

| Chromosome | Average distance (MB*) | Average r^2^ | Number of markers |
| --- | --- | --- | --- |
| 1 | 4,043.72 | 0.40 | 39,121 |
| 2 | 4,121.77 | 0.40 | 33,155 |
| 3 | 4,046.88 | 0.40 | 29,995 |
| 4 | 4,080.57 | 0.41 | 29,556 |
| 5 | 4,204.40 | 0.43 | 28,815 |
| 6 | 3,870.82 | 0.42 | 30,552 |
| 7 | 4,037.92 | 0.41 | 27,617 |
| 8 | 4,680.89 | 0.38 | 24,207 |
| 9 | 4,072.88 | 0.42 | 25,942 |
| 10 | 3,933.11 | 0.39 | 26,508 |
| 11 | 3,852.74 | 0.41 | 27,833 |
| 12 | 4,180.11 | 0.40 | 21,791 |
| 13 | 4,803.52 | 0.36 | 17,510 |
| 14 | 4,498.18 | 0.39 | 18,531 |
| 15 | 4,056.76 | 0.39 | 21,009 |
| 16 | 3,965.15 | 0.42 | 20,394 |
| 17 | 3,852.67 | 0.41 | 19,370 |
| 18 | 3,877.15 | 0.41 | 16,982 |
| 19 | 3,857.93 | 0.38 | 16,577 |
| 20 | 3,805.86 | 0.40 | 18,898 |
| 21 | 4,053.16 | 0.40 | 17,653 |
| 22 | 3,797.84 | 0.41 | 16,123 |
| 23 | 3,914.24 | 0.37 | 13,402 |
| 24 | 3,999.31 | 0.41 | 15,638 |
| 25 | 3,770.64 | 0.37 | 11,344 |
| 26 | 3,818.05 | 0.39 | 13,501 |
| 27 | 3,879.77 | 0.38 | 11,729 |
| 28 | 4,008.62 | 0.39 | 11,536 |
| 29 | 4,030.92 | 0.38 | 12,694 |

*Mega base pairs; Data set no MAF applied.
